# Online Hydrogen-Deuterium Exchange Traveling Wave Ion Mobility Mass Spectrometry (HDX-IM-MS): a Systematic Evaluation

**DOI:** 10.1007/s13361-017-1633-z

**Published:** 2017-04-03

**Authors:** Adam Cryar, Kate Groves, Milena Quaglia

**Affiliations:** 0000 0004 0556 5940 grid.410519.8 LGC, Queens Road, Teddington, London, TW11 0LY UK

**Keywords:** Hydrogen-deuterium exchange mass spectrometry, Ion mobility, Data-independent acquisition, MS^E^, Protein structure

## Abstract

Hydrogen-deuterium exchange mass spectrometry (HDX-MS) is an important tool for measuring and monitoring protein structure. A bottom-up approach to HDX-MS provides peptide level deuterium uptake values and a more refined localization of deuterium incorporation compared with global HDX-MS measurements. The degree of localization provided by HDX-MS is proportional to the number of peptides that can be identified and monitored across an exchange experiment. Ion mobility spectrometry (IMS) has been shown to improve MS-based peptide analysis of biological samples through increased separation capacity. The integration of IMS within HDX-MS workflows has been commercialized but presently its adoption has not been widespread. The potential benefits of IMS, therefore, have not yet been fully explored. We herein describe a comprehensive evaluation of traveling wave ion mobility integrated within an online-HDX-MS system and present the first reported example of UDMS^E^ acquisition for HDX analysis. Instrument settings required for optimal peptide identifications are described and the effects of detector saturation due to peak compression are discussed. A model system is utilized to confirm the comparability of HDX-IM-MS and HDX-MS uptake values prior to an evaluation of the benefits of IMS at increasing sample complexity. Interestingly, MS and IM-MS acquisitions were found to identify distinct populations of peptides that were unique to the respective methods, a property that can be utilized to increase the spatial resolution of HDX-MS experiments by >60%.

**Electronic supplementary material:**

The online version of this article (doi:10.1007/s13361-017-1633-z) contains supplementary material, which is available to authorized users.

**Graphical Abstract:**
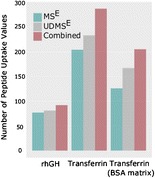
ᅟ

## Introduction

Better characterization of higher order protein structure which encompasses secondary, tertiary and quaternary structures, is an important element to aid the understanding of the link between protein structure and biological function. Protein conformational ensembles are not only determined by primary sequence but also by a variety of factors which include protein interactions, post-translational modifications and local cellular environment [1–4].

Hydrogen-deuterium exchange mass spectrometry (HDX-MS) uses the ability to reversibly modify protein backbone amide hydrogens with deuterium to probe conformational dynamics [5, 6]. By incubating a protein in high levels (>90%) of deuterium oxide (D_2_O) for defined periods of time, labile hydrogens are exchanged with deuterium. The rate at which exchange occurs at each residue is directly related to protein structure and can therefore be used to monitor protein dynamics, sites of interaction and the effects of protein modifications across experimental timescales ranging from seconds to hours [7–9]. The use of mass spectrometry to measure the extent of deuteration, where the resultant mass shift in the observed protein or peptide isotopic envelope is a direct measurement of the extent of exchange, means that sample requirements are minimal in comparison to other established structural techniques [10, 11].

HDX-MS experiments can either be performed without a proteolytic digestion step, which provides global structural information, or with the addition of a digestion step which produces more localized structural data. Due to the reversible nature of the HDX process a major technical caveat to both of these experiments is the need for all steps post-exchange to be performed at conditions which best quench the exchange process thereby improving the ability of the method to detect small conformational differences [12, 13]. These conditions are low pH and low temperatures with short analytical timeframes also typically used. It is for this reason that protein digestion in local HDX-MS experiments is performed with an acid-tolerant enzyme prior to analysis by liquid chromatography mass spectrometry (LC-MS). As previously mentioned this process facilitates the determination of local resolution structural information through the measurement of peptide specific exchange rates [14]. Pepsin is the favoured enzyme, due to its high activity at low pH and low cleavage site specificity.

The spatial resolution afforded by a HDX-MS experiment is dependent on the number of proteolytic peptides that can successfully be identified. By maximising the combination of protein sequence coverage and overlapping peptides detected (peptide redundancy) HDX-MS experiments can produce local structural information across almost complete protein sequences [13, 15, 16]. Recently the use of electron transfer dissociation (ETD) has been shown able to increase HDX-MS resolution down to single amino acid residue localization, however, this methodology is not yet widely applied [17–19]. It has also been previously demonstrated that when peptide redundancy is sufficiently high the correct computational method can provide amino acid residue exchange rates without the need for peptide fragmentation [20]. Here peptide redundancy refers to the number of detected peptides that describe the deuterium uptake of an amino acid residue, averaged over the complete protein sequence.

Local structural HDX data are relatively straightforward to acquire for small proteins but as protein size and sample complexity increase chromatographic limitations enforced to minimize back-exchange can hinder peptide identification and hence structural resolution. Even upon the successful identification of a proteolytic peptide exchange data can be rendered unusable during downstream analysis by the presence of overlapping isotopic distributions from co-eluting, and almost isobaric peptides. This problem of overlapping isotopic distributions is compounded by the expansion of such distributions upon the exchange of hydrogen with deuterium where isotopic envelopes can subsequently span a *m/z* range of >5.

Traveling wave ion mobility spectrometry (TWIMS), which separate gas phase ions by cross sectional size and charge, was incorporated into commercial instruments more than ten years ago [21]. Recently, TWIMS has been adapted to allow the separation of complex peptide mixtures on the millisecond timescale and has been integrated into commercial instruments including the Synapt G2Si [22, 23]. The additional dimension of separation, unlike more established chromatographic methods, is able to increase system peak capacity without additional analysis time [24]. This is of particular benefit to HDX-MS experiments where increased analysis times will often translate to increased levels of back-exchange. It has been previously shown that when using the data independent acquisition (DIA) approach, MS^E^ [25], the addition of IMS (HDMS^E^) can increase peptide identifications from complex protein digests by > 50% [26]. This method was optimized by U. Distler and co-workers to allow optimal precursor collision energies to be estimated based on measured arrival times (UDMS^E^) [27]. It was shown that this approach rather than the single collision energy ramp that is used during a HDMS^E^ acquisition provided higher peptide fragmentation efficiency. Using optimized collision energies, they successfully demonstrated a further increase in peptide identifications of ~50%.

Data has been previously published demonstrating that IMS, coupled to an online HDX-MS system, increases system peak capacity with high reproducibility[28, 29]. A systematic evaluation of online hydrogen-deuterium exchange ion mobility mass spectrometry (HDX-IM-MS) on current QToF instrumentation has not yet, however, been reported. Here we address the use of TWIMS on a Synapt G2Si coupled to an automated online-HDX system at increasing sample complexities and its effects on experimental structural resolution and measurement reproducibility. Included herein is, to our knowledge, the first reported application of UDMS^E^ to HDX analysis.

## Experimental

### Materials and Methods

All chemicals were purchased from Sigma Aldrich (Gillingham, UK) unless otherwise stated. TCEP and formic acid were purchased from Thermo Fisher (Hemel Hempstead, UK), acetonitrile was Optigrade HPLC Special Grade (LGC Standards, Teddington, UK), and ultra-pure water (18 MΩ cm^–1^) was used.

### Sample Preparation

Recombinant human growth hormone (rhGH) reference standard was purchased from the WHO (WHO98/574, NIBSC, Potters Bar, UK), whilst human transferrin (Apo and Holo form) and bovine serum albumin (BSA) were purchased from Sigma Aldrich. Proteins were solubilized in 10 mM phosphate buffer at pH 7.4 and pH 7.0, respectively. The rhGH stock was prepared at 0.8 mg/mL whilst the human transferrin and BSA stocks were solubilized at 2.0 mg/mL.

Control samples were diluted in 10 mM phosphate buffer (1 in 5 dilution). Zinc bound rhGH (rhGH:Zn) was prepared by dilution in 90 μM zinc acetate. Apo- and holo-transferrin samples were prepared by dilution with 10 mM phosphate + 3.125 mM ammonium bicarbonate. For the transferrin + BSA samples, equimolar amounts of the transferrin and BSA stocks were mixed and subsequently diluted with 10 mM phosphate buffer to a final dilution of 1 in 5.

### Hydrogen-Deuterium Exchange and Online Digestion

Sample handling and mixing were performed using a LEAP PAL system set (LEAP Technologies, Carrboro, NC, USA). For each run, a 15 μL protein sample aliquot was diluted 1 in 10 in 10 mM phosphate buffer (pH 7.4 or pH 7.0) prepared in either H_2_O or D_2_O. For D_2_O exchange experiments, samples were incubated at room temp for 2.30, 60, or 240 min. The exchange lengths included were chosen based on the known structural dynamics of the two model proteins. This information had been previously determined using HDX-MS experiments with five time-points that ranged from 30 s to 8 h. Sample dilution with H_2_O buffer was used for either peptide map generation or as a 0 min reference.

Hydrogen exchange was quenched using a 1:1 dilution in 100 mM phosphate buffer, pH 2.5. For rhGH, the quench solution contained 2 M guanidine hydrochloride (GndHCl) and 300 mM tris-(2-carboxyethyl)phosphine (TCEP), whilst for transferrin the solution contained 200 mM TCEP only. Quench solutions had been previously optimized for efficient pepsin digestion. Samples were quenched at 4 °C for 30 s before being loaded onto an Enzymate BEH 5 μm pepsin column, 2.1 × 30 mm (Waters, Manchester, UK). Digestion temperature was maintained at 25 °C and 15 °C for rhGH and transferrin, respectively. Both proteins were digested at a flow rate of 70 μL/min and a pressure of ~8000 psi. Pressure was maintained during digestion through the use of a peek restrictor placed just prior to the waste line [30].

### LC-MS

LC-MS/MS analyses were performed using a Synapt G2Si (Waters, Manchester, UK) coupled to a nano-ACQUITY with HDX technology (Waters, Manchester, UK).

Post-pepsin digestion peptides were loaded onto an ACE C18 guard cartridge (HiChrom, Reading, UK, 5 μm, 2.1 mm) and desalted. Peptides were chromatographically separated on a ACE Excel SuperC18-AR (2.1 × 150 mm, 2 μm; HiChrom, Reading, UK) using a linear gradient starting at 8% acetonitrile, 0.1% FA, and increasing over 7 min to 35% with a flow rate of 100 μL/min. All chromatographic steps, including desalting, were carried out at 0 °C.

Mass spectrometric-based peptide maps were generated with the instrument operated in the DIA modes MS^E^, HDMS^E^, and UDMS^E^. For all measurements, the quadrupole was used in rf-only mode, with the quadrupole tuned such that only ions with *m/z* higher than 300 were transmitted. For MS^E^ and HDMS^E^ experiments, collision energy within the trap was continuously alternated between low energy (MS data) and a high energy ramp (MSMS data) throughout the chromatographic run.

For HDMS^E^ and UDMS^E^ measurements the instrument was operated in ion mobility mode. The T-wave was operated with a wave height of 40 V and a wave velocity ramp from 500 to 800 m/s. The values used were identical to those used by Distler and colleagues to ensure comparability [27]. Comparability of IMS was confirmed by the monitoring the drift time of [Glu1]-fibrinopeptide B peptide fragment ions prior to analysis. For UDMS^E^ measurements applied collision energies were calculated based upon an ions arrival time where energies were specified in a.luc file. Collision energies were as previously taken from the work of Distler and co-workers [27]. Deuterium exchange measurements were made in either MS or IM-MS mode with ion mobility settings unchanged from UDMS^E^ measurements.

For all experiments, time of flight measurements were made in sensitivity mode with a typical resolving power of >18,000. A scan time of 0.6 s was used for all measurements and data was post-acquisition lock mass-corrected using the 2+ charge state of [Glu1]-fibrinopeptide B, which was infused at a concentration of 100 fmol/μL at 90° to the analytical sprayer.

### Data Processing

DIA data were processed in ProteinLynx Global Server (PLGS) ver. 3.02 (Waters﻿, Manchester, UK). PLGS processing steps prior to database searching are detailed elsewhere [25]. Briefly, data were centroided, de-isotoped, and charge state-reduced prior to the tentative assignment of fragment ions to parent proteins based upon retention time alignment. Peak picking thresholds for MS^E^ data were 500 counts for low energy, 100 counts for high energy, and 750 counts for precursor exact mass retention time (EMRT) integrated intensity. For HDMS^E^ and UDMS^E^, lower thresholds were used: 165 counts, 33 counts, and 250 counts. Peak lists were database-searched against either the Uniprot somatotropin sequence (P01241) or the human transferrin sequence (Q06AH7). In both cases, fasta files were appended with the amino acid sequence of porcine pepsin (P00791). Precursor and fragment ion mass tolerances were set to 10 and 20 ppm, respectively, and enzyme specificity was set to nonspecific. Protein identification criteria were set as the detection of at least two fragments per protein, seven fragments per peptide, and at least one peptide per protein. Oxidation of methionine was set as the only variable modification. The protein level false discovery was maintained below 2% with the use of a reversed database.

PLGS search results and deuterium exchange measurements were imported into DynamX ver. 3.0 (Waters, Manchester, UK ). For a peptide to be retained within the peptide map, it must have been identified in four out of five LC-MS/MS injections, have a minimum of 0.01 products per amino acid, and have a precursor mass error of less than 20 ppm as defined within DynamX. Deuterium exchange measurements were analyzed with default settings and all data were manually validated and curated if required.

## Results and Discussion

### Peptide Map Generation: MS^E^, HDMS^E^, UDMS^E^

**Figure 1:**
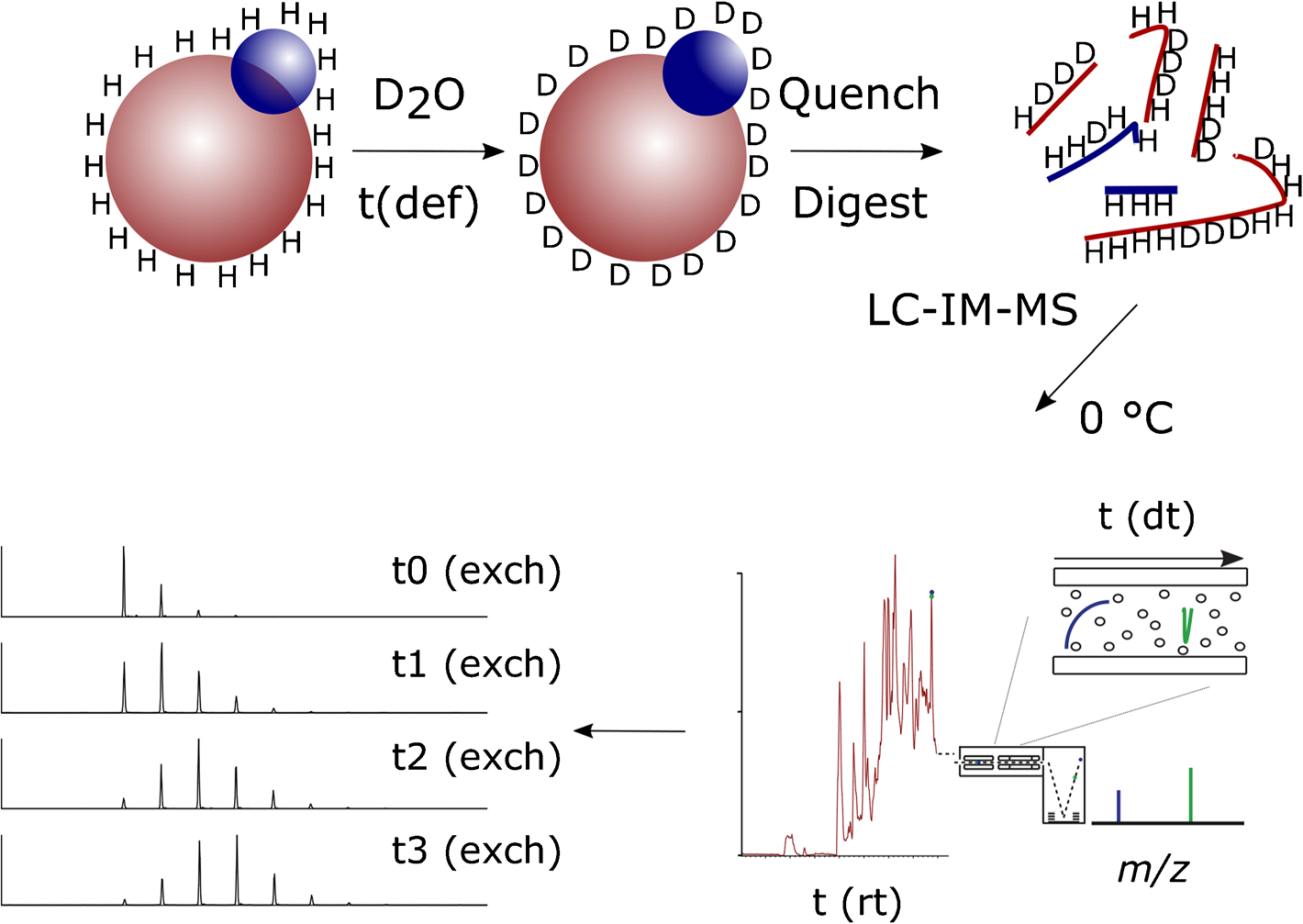
Schematic of a typical online hydrogen-deuterium exchange mass spectrometry experiment and how ion mobility can be successfully integrated into the workflow

Due in part to the commercialization of TWIMS for peptide separation and the relatively recent development of propriety HDX-MS processing software that is IM-MS compatible, HDX-IM-MS is more straightforward and requires less specialist knowledge than when first reported. Although not a high resolution separation method, the millisecond time-scales within which the technique operates and its orthogonality to RP-LC means that IMS is gaining popularity as a separation method for peptide analysis. How IMS fits into a typical online-HDX-MS method is shown in Figure 1.

**Figure 2:**
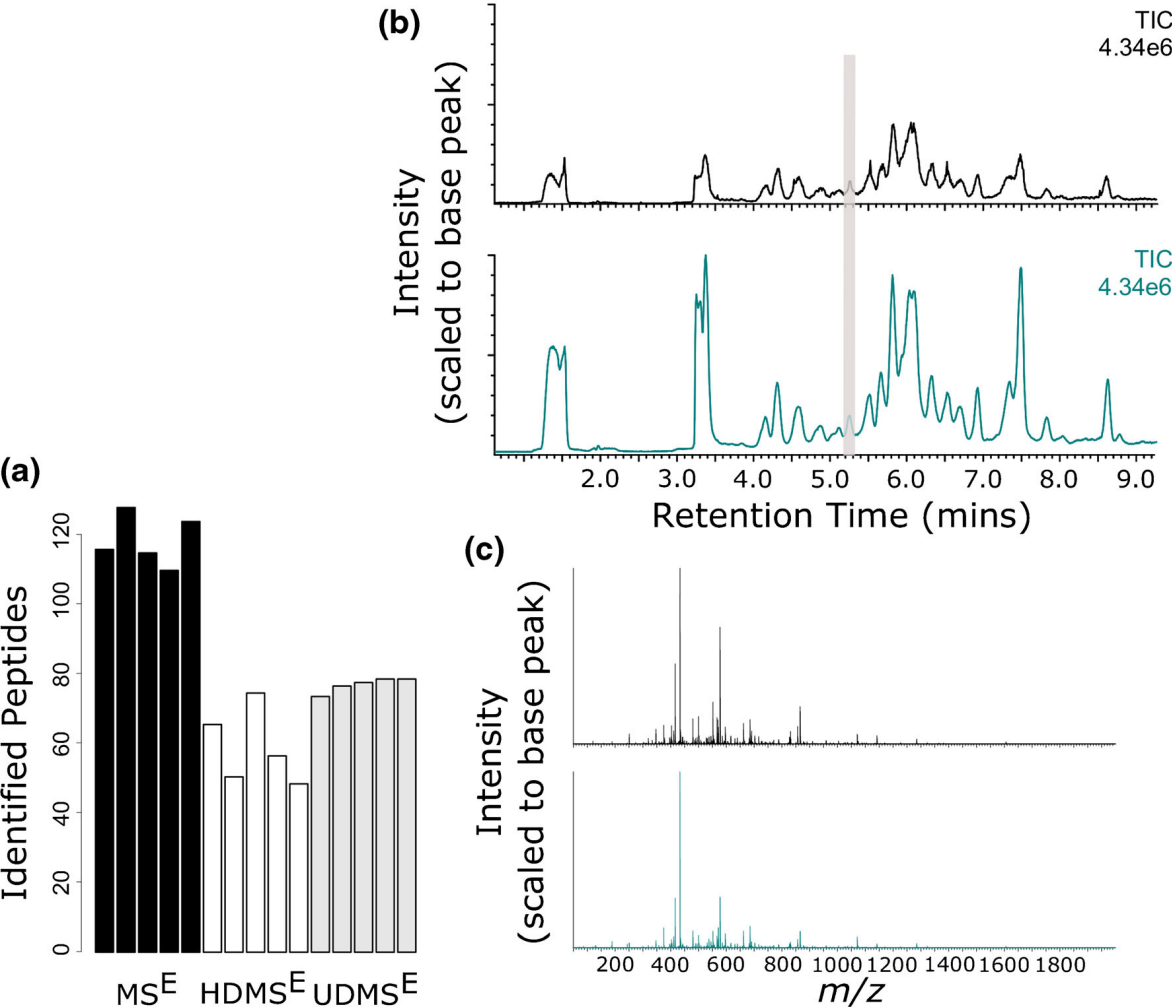
rhGH peptide maps from three different DIA modes of acquisition. (**a**) Number of rhGH peptide identifications in MS^E^, HDMS^E^, and UDMS^E^ modes. (**b**) Typical TIC chromatograms from the analysis of rhGH in MS^E^ (Cambridge blue) and UDMS^E^ (black) modes. (**c**) A comparison of MS^E^ vs UDMS^E^ spectral intensities shows detector saturation of the highest intensity ions. Data shown has been combined across the retention time window highlighted in Figure 1b. MS^E^ and UDMSE measurement are shown in Cambridge blue and black, respectively. Lower abundance ions can be seen to be proportionally (in reference to the base peak) more intense when in UDMS^E^ mode (shown in black). This is the result of the highest intensity ions being under-represented due to detector saturation

An initial assessment of the implementation of ion mobility within a HDX-MS workflow was performed on recombinant human growth hormone (rhGH). rhGH is a pituitary hormone that has been previously used in our lab as a model system for optimizing and assessing HDX-MS reproducibility and sensitivity (K. Groves et al., “Systematic evaluation of the effects of sequential HDX workflow parameters on measurement reproducibility,” manuscript in preparation (2017)). Using digestion conditions that had been previously determined to produce a sequence coverage of >90% of the number of peptides successfully identified in MS^E^, HDMS^E^ and UDMS^E^ modes were compared, Figure 2a. As expected, UDMS^E^ outperformed HDMS^E^ in terms of the number of total peptide identifications. Examination of high energy data (analogous to DDA MS/MS spectra) found UDMS^E^ to produce more efficient peptide fragmentation than HDMS^E^, which is in agreement with previous literature, Supplementary Figure 1a [27]. An example comparison of HDMS^E^ and UDMS^E^ peptide fragmentation spectra is provided in Supplementary Figure 1b. Despite the enhancement in fragmentation efficiency, it was the MS^E^ mode of acquisition that identified the largest number of peptides, Figure 2a and Supplementary Figure 2. These data were in contrast to all current MS peptide analysis literature that reports improved peptide identifications upon ion mobility implementation [31, 32]. Comparative analysis of TIC traces identified a 4-fold reduction in measured intensity upon the implementation of mobility separation, Figure 2b. Detector saturation, resulting from the peak compressing effects of IMS, has been previously reported to negatively affect protein quantification in HDMS^E^ proteomic data [33]. This has not, however, been previously observed to reduce peptide identification rates in comparison to MS^E^ measurements. We hypothesized that due to the large amounts of single protein solutions that are loaded on column (low pmol range) during a HDX-MS experiment, large scale detector saturation may be causing a reduction in measured peptide intensity, thereby limiting peptide identifications. Indeed, when spectra containing high intensity ions were compared between HDMS^E^ and MS^E^, it was apparent that ion mobility implementation caused the true intensity of ions to be under represented, Figure 2c.

The Synapt G2Si allows for the extension of dynamic range through the application of a dynamic range extension (DRE) tool. When this mode is activated, dynamic range is extended through the acquisition of two separate scans in immediate succession; a normal scan and a signal attenuated scan. These data are computationally stitched together to produce pseudo single scan data, which can be submitted for database searching. The downside of this approach is that scan speeds are rendered twice as long, thus reducing the number of measurement points across a peak. Implementation of DRE (50:50 split between attenuated and non-attenuated) was found to increase the number of peptide identifications by 30% and 60% for HDMS^E^ and UDMS^E^, respectively. Additionally, it was observed that the use of DRE increased TIC signal intensity by around 50% in comparison to normal UD/HDMS^E^ modes. This confirmed that a large proportion of the observed drop in intensity can be attributed to detector saturation. The observation was also corroborated by an observed increase in relative intensity for higher abundance ions when DRE mode was switched on. For all subsequent experiments, UDMS^E^ and IM-MS analyses were performed with DRE activated. It is important to note that the observed levels of detector saturation versus amount of protein loaded should be considered as an important factor when acquiring peptide map information and performing HDX-MS experiments. Our data suggests that the point of saturation is dependent on the MS settings used. Although the use of DRE is currently the only method available on the Synapt G2Si to overcome IMS-related detector saturation, other procedures have been documented, the use of which might help circumvent the issues of saturation in HDX-IM-MS measurements. Notably, the multiplexing of ion injections into the drift cell or the gating of the pre-trap cell, to allow a variable trapping time, could both be beneficial if implemented into the current acquisition methodology [34, 35].

Despite a reduction of signal intensity upon the use of IMS, data has been previously published demonstrating the ability of IM-MS to provide higher sensitivities than MS alone. This is attributed to the removal of interfering background chemical noise or co-eluting ions [36]. Despite a reduction in signal intensities, examination of our data observed an increase in signal to noise for the majority of ions tested that were not subject to detector saturation. During data processing, we wished to account for the differences in signal intensity and signal to noise between MS and IM-MS data. To do this, peak picking thresholds used for IM-MS data were reduced from those that had been previously determined to produce reproducible peptide identifications from MS^E^ replicate data. Following these changes, we observed that an equivalent number of peptides were identified between MS^E^ and UDMS^E^ whereas HDMS^E^ still identified fewer.

Following the optimization of a UDMS^E^ method suitable for HDX-MS peptide map generation, we investigated how MS and IM-MS derived peptide maps compare at three different sample complexities. rhGH was tested as an example of a sample with low complexity, human transferrin for medium complexity, and finally an equimolar solution of transferrin and bovine serum albumin (transferrin + BSA) was tested as a proxy for samples of higher complexity (combined molecular mass of 146 kDa). It was found that marginally fewer peptides were identified with the UDMS^E^ method, suggesting that peak capacity is not a limiting factor for peptide identification, when analyzing small protein systems. UDMS^E^ and MS^E^ provided comparable peptide identifications for transferrin, whilst a minor improvement (~7% increase) in the total number of transferrin peptides identified was only present for the sample of highest complexity, transferrin + BSA. Detailed results are provided in Table 1. Our peptide map data, therefore, confirms that UDMS^E^ is of limited use for the peptide map generation of small proteins but that the increase in peak capacity is of benefit for more complex systems; in our case ≥140 kDa.

**Table 1:** Total Number of Peptides Retained for HDX Peptide Maps as Identified by MS^E^ and UDMS^E^ Analysis. Peptides were Filtered from Database Search Results Prior to Peptide Map Generation Based on Replication, Presence of Fragments, and Mass Accuracy

Sample	MS^E^ N^o^ peptides	MS^E^ Coverage (%)	MS^E^ redundancy	UDMS^E^ N^o^ peptides	UDMS^E^ Coverage (%)	UDMS^E^ Redundancy	MS^E^ UDMS^E^ Common peptides
rhGH	99	95.8	6.23	93	97.9	5.50	74
Transferrin	270	95.7	6.12	271	91.0	6.49	175
Transferrin (BSA matrix)	198	94.8	5.11	221	90.3	5.20	109

**Figure 3:**
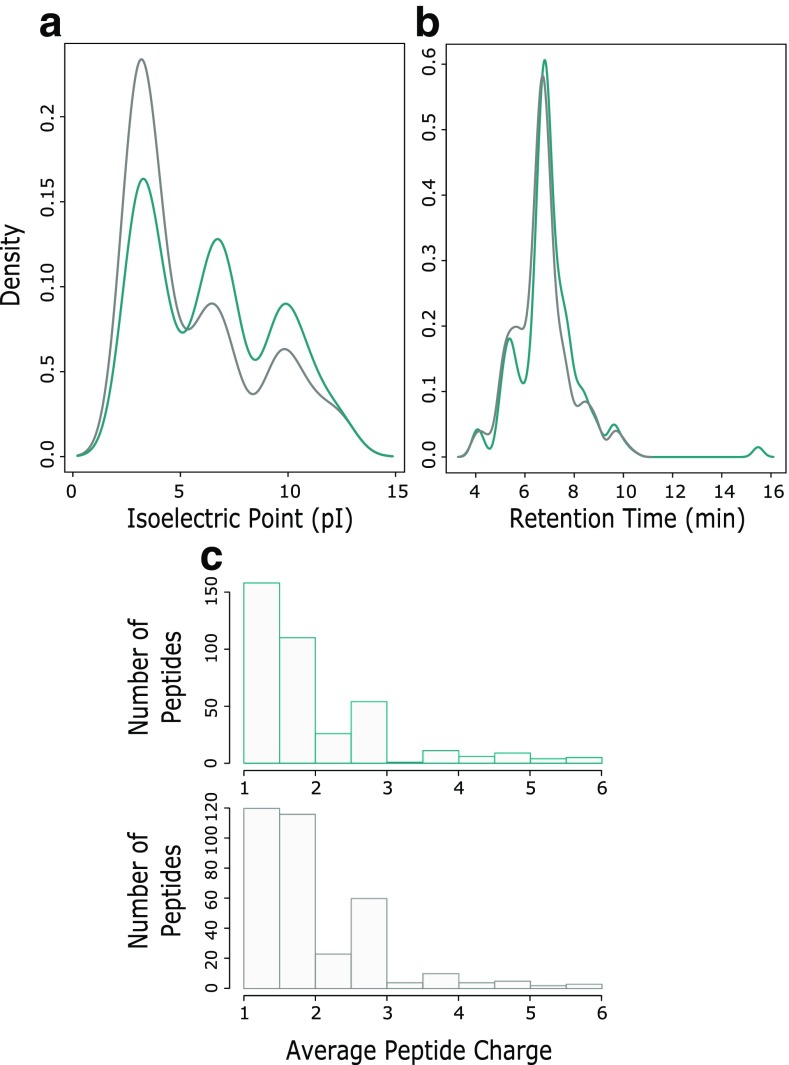
Comparison of the physicochemical properties of rhGH proteolytic peptides identified by MS^E^ and UDMS^E^ analysis, shown in Cambridge blue and grey, respectively. (**a**) Density plot of peptide pI. (**b**) Density plot of peptide retention times. (**c**) Histogram of average peptide charge as calculated by PLGS

Interestingly, MS^E^ and UDMS^E^ were found to identify a considerable proportion of peptides that were unique to the respective acquisition methods. A possible explanation for this is the different approaches the two methods use to apply fragmentation energy to eluting peptides. If this were true, no physicochemical bias should be present when comparing the peptide identified. To test for any signs of bias between the two methods, identified peptides from the transferrin dataset (MS^E^ and UDMS^E^) were compared for peptide retention time, charge, and isoelectric point, Figure 3. Limited differences were observed, confirming no detectable inherent biases were present for the two methods.

### HDX-IM-MS

**Figure 4:**
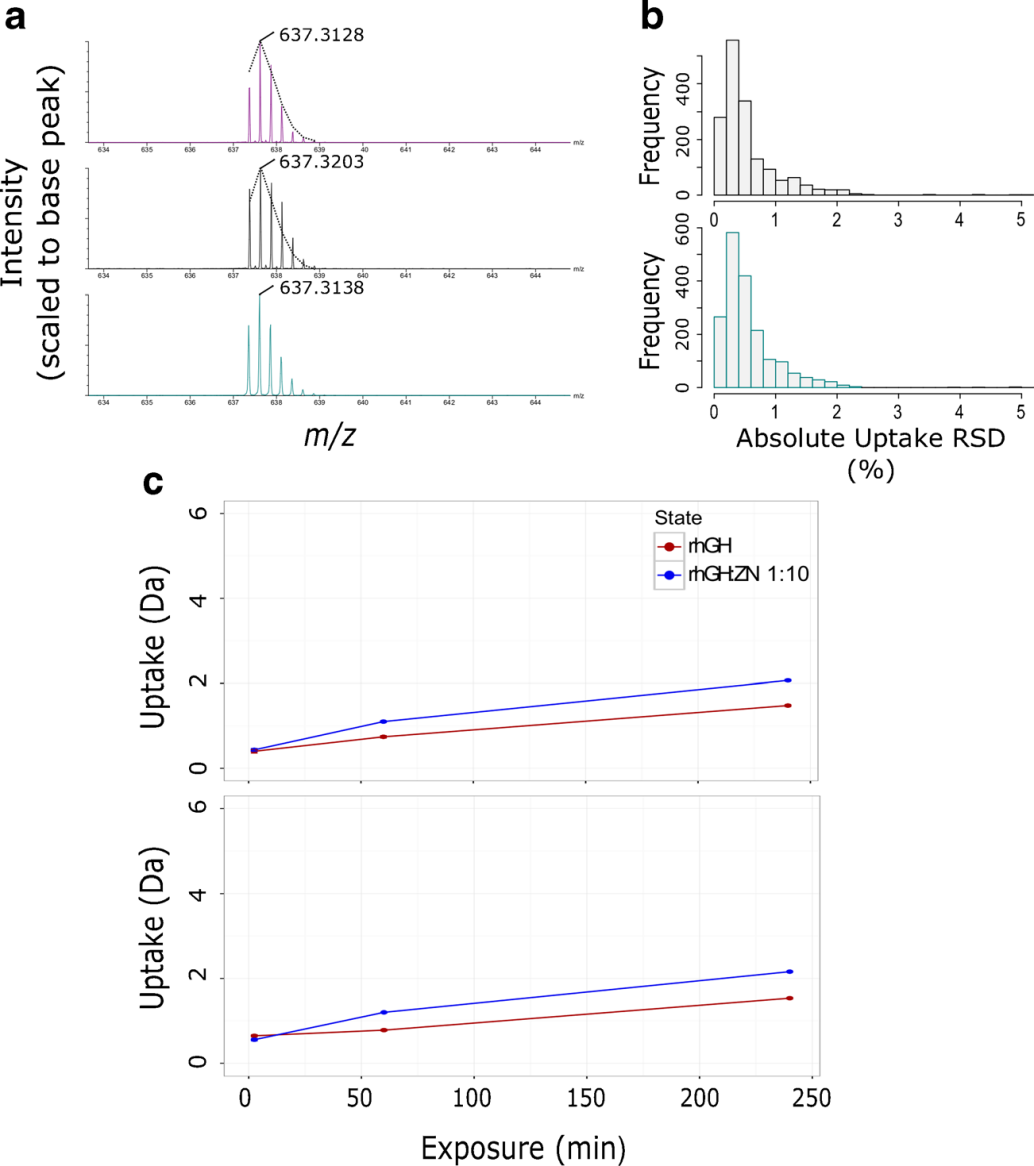
Direct comparisons of rhGH HDX time-course data acquired in MS and IM-MS modes shows data to be comparable. (**a**) Example of detector saturation as a result of mobility-induced peak compression and the effects on observed isotopic distribution and mass measurement. Data shown is the same peptide ion measured in MS (Cambridge blue), IM-MS (black), and IM-MS mode with DRE (purple). The overlaid dotted black lines are the isotopic envelope as measured by MS for comparison with IM-MS spectra. Labeled *m/z* values are after lockmass correction. (**b**) Histogram of calculated rhGH peptide absolute deuterium incorporation standard deviations (n = 5). Data from MS and IM-MS modes are shown in Cambridge blue and grey, respectively. **(c)** Example rhGH peptide uptake plots from a four-point time-course exchange experiment with data acquired in MS (top) and IM-MS (bottom) modes. Data was collected for rhGH (red) and rhGH incubated with a ×10 excess of zinc acetate (blue). Data shown are averages of repeat measurements (n = 3). Error bars show standard deviation of repeat measurements

Following peptide map generation, HDX data acquisition is predominantly performed in MS mode only. Peptide identifications are inferred based on accurate mass and retention time thresholds. Although modern LC-QToF instrumentation offers high resolution (typically >20,000) [37, 38] and excellent retention time stability [39], these two properties may not always be sufficient to distinguish co-eluting ions of the same nominal mass. Ion mobility provides an extra property based on which peptide identifications can be transferred across to MS HDX data. We confirmed TWIMS to be highly reproducible across triplicate injections, where the median coefficient of variation was found to be 0.55%. This, therefore, makes it an ideal measurement that can provide further specificity to HDX data processing. Within our IM-MS data, however, and where peptide ion signal saturation was most acute, we observed an alteration in measured isotopic distributions, Figure 4a. The frequency and significance of this observation was reduced by the implementation of DRE but not totally removed. Where a change in isotopic distributions was observed, it was accompanied by a bias towards increased measured *m/z* values. We found this bias to be partially reduced by lockmass correction. Mass measurement errors from MS^E^ and UDMS^E^ data post-lockmass correction are compared in Supplementary Figure 3.

HDX measurements are based entirely on isotopic distributions. We, therefore, wanted to determine whether the observed effects of signal saturation caused significant differences in measured deuterium uptake levels in comparison to our reference MS measurements. As previously reported [29], HDX uptake measurements for rhGH were found to be comparable when acquired in MS and IM-MS modes. MS and IM-MS calculated uptake values had a median difference of only 2.15% based on absolute uptake values. Measurement precision was also found to be similar with median coefficient of variations being 1.5% and 2.5% for MS and IM-MS data, respectively, Figure 4b. A similar 1% difference in measurement precision was observed in all data sets generated from this assessment. Example uptake values for a typical rhGH peptide measured in both MS and IM-MS modes are shown in Figure 4c.

**Figure 5:**
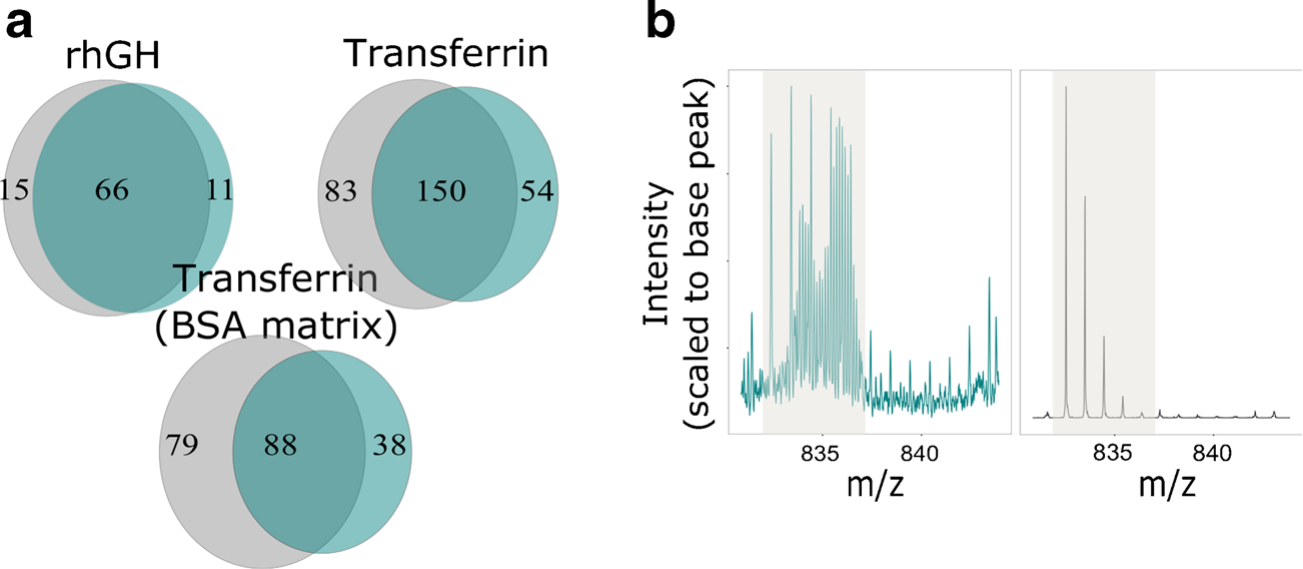
(**a**) Total number of peptide uptake values successfully calculated from three, four-point, HDX time-course experiments when acquired in MS (Cambridge blue) and IM-MS (grey) modes. Peptide were removed from initial peptide maps if ion interferences were present or the identity of the ion could not be confirmed based on RT, *m/z*, and arrival time (if available). (**b**) Example of the IMS separating interfering ions of a different charge state from the peptide ion of interest, allowing the calculation of accurate uptake values. MS data (blue) and IM-MS data (black) shown have been extracted within DynamX using identical settings. The area highlighted in grey denotes the expected *m/z* region for the undeuterated peptide ion

Despite often having high quality fragmentation data, peptides identified by LC-MS are not always suitable for deuterium uptake calculation as overlapping isotopic distributions of deuterated peptides can prevent the accurate calculation of uptake levels. IMS is able to effectively separate peptides that are detected with almost identical *m/z* but are of a different charge state or cross-sectional size. This extra degree of separation should reduce the number of peptides that are subject to interferences and increase the number of peptides for which uptake values can be calculated. We postulated that the occurrence of overlapping distributions would be more frequent as the size of the protein being studied increases. The benefits of IMS should, therefore, be more pronounced as sample complexity increases. To test this, we performed a series of four-point time-course exchange experiments for rhGH, transferrin, and transferrin + BSA. For each time-course analysis, we included two different conditions (see materials and methods) to best mimic the complexity of a typical HDX experiment. Results for the three datasets after manual validation of the data are shown in Figure 5a. Little difference is observed between MS and IM-MS results for the smallest protein tested, rhGH, confirming that sample complexity is low enough that LC-MS alone provides enough peak capacity to fully resolve the majority of detectable peptides. A 14% increase in peptides with measureable uptake values were obtained when using IM-MS (versus MS) for HDX time-course analysis of transferrin, providing evidence that IM separation becomes beneficial when studying medium-sized proteins. As expected, we found that IMS produced the largest increase in peptide uptake data for the most complex sample tested, transferrin + BSA. Analysis of these data demonstrates that if transferrin peptides only are considered, IM-MS analysis is able to provide deuterium uptake values for 41 more peptides than MS mode alone. This represents a 33% increase on the traditional analytical approach. Although BSA-derived peptides in these data were not subject to time-course analysis (and therefore manual validation) within DynamX, if all peptides identified for transferrin and BSA are considered, the data can be used as a proxy for a protein with a molecular mass ~146 kDa. Based on the total number of peptides that were reproducibly identified by the two modes, Supplementary Figure 4, and the percentage of transferrin peptides that were removed during manual curation, our data can be used to estimate that the use of IM-MS over MS would yield deuterium uptake values for an extra 99 peptides for a protein size that is comparable to that of an antibody.

An example of how ion mobility separation can enhance HDX-MS measurements is shown in Figure 5b. Here ion mobility effectively removes interfering peptide ions that are of a different charge state to the peptide ion of choice. Without the use of ion mobility it is not possible to obtain uptake values for this peptide and the data must be removed during the manual validation process. Our evaluation has demonstrated that at least for proteins <146 kDa, the benefits of ion mobility become substantially larger after the data has been manually curated and peptides with overlapping isotopic distributions removed.

**Figure 6:**
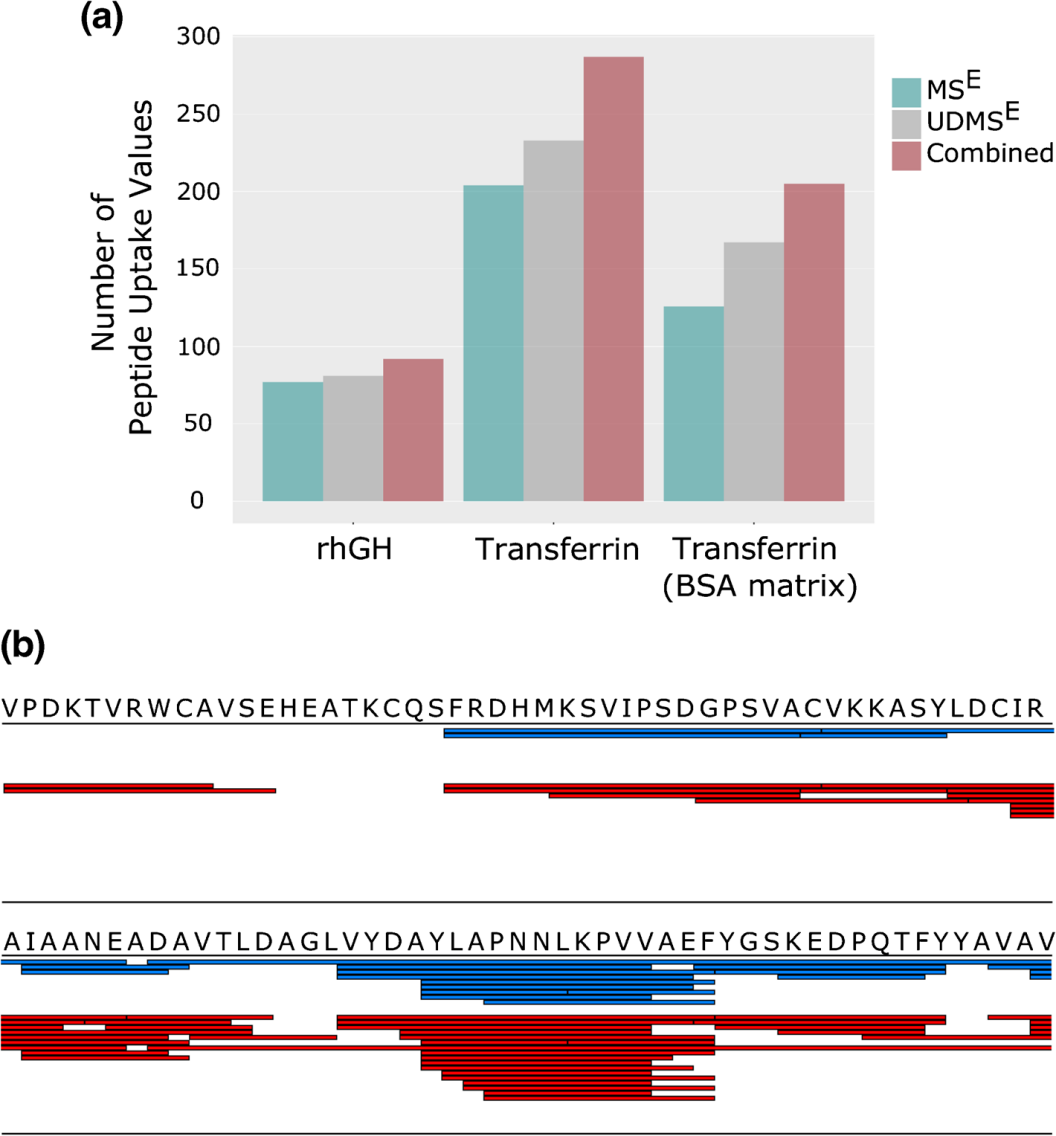
Results from HDX time-course analyses where MS and IM-MS HDX acquisition methods have been used in parallel and combined post-processing. (**a**) Total number of calculated peptide deuterium uptake values (after manual validation) for the three different model protein samples tested. Compared are the results from MS^E^ analysis, UDMS^E^ analysis, and from a combination of both acquisition methods. (**b**) Overlaid sequence coverage maps (after manual validation of time-course data) when acquiring data in MS^E^ mode (blue) or using the combined approach (red) for the transferrin (BSA matrix) sample. Shown is a 100 amino acid sequence section of transferrin that is representative of the full data set (found in Supplementary Figure 5)

In cases where the structural resolution of a HDX experiment is of upmost importance, it is possible to utilize the observation that MS and IM-MS acquisitions identify unique subpopulations of peptides. By acquiring data in both MS and IM-MS modes, within the same time-course experiment, and combining the results post hoc, it is possible to increase the number of measured uptake values by >60%, Figure 6a. For this approach, a script was written in the statistical programming language R, Supplementary Data, to combine the results from MS and IM-MS analyses. Our data again confirms that the benefits of this approach are correlated to the complexity/size of the protein being studied.

It is important to note that the aforementioned approach led primarily to larger gains in peptide redundancy as opposed to sequence coverage, Figure 6b and Supplementary Figure 5. For proteins of a size within the range of those tested in this evaluation, our data provides evidence that the optimization of the digestion conditions is the key component in obtaining high sequence coverage. Optimization of the mass spectrometry method is, on the other hand, a more important element for maximizing peptide redundancy and, therefore, the spatial resolution of the technique. It is likely, however, that for larger proteins (>150 kDa) where >90% sequence coverage will prove more difficult to obtain with LC-MS alone due to a much larger sample complexity, the implementation of IM-MS would also dramatically increase protein coverage results.

An apparent downside to this process is the increase in the time required for both data acquisition and processing. Despite this increase, analyses times do still compare favorably with other analytical approaches that have been previously reported to increase the spatial resolution of HDX-MS results, where manual changes to instrument configurations are required [14, 40]. Indeed, for larger proteins such as antibodies, where required levels of characterization and the increase in calculated uptake values are likely to both be high, the resultant data will often merit the increased analysis times.

## Conclusions

HDX-MS offers low levels of sample requirement and versatility that the ‘gold standard’ structural techniques NMR and X-ray crystallography cannot. Although a lower spatial resolution technique than the afore-mentioned, the evolution of HDX-MS over the past 20 years, through software and instrumentation developments, has and will continue to push forward the resolution that can be achieved [41–43]. Despite the integration of ion mobility within commercially available HDX systems having been previously presented, the benefits and how to successfully optimize data acquisition are not immediately evident. Our evaluation provides evidence that the integration of TWIMS within established online-HDX-MS workflows, HDX-IM-MS, can provide improved system peak capacity on-the-fly and accurate deuterium uptake measurements but the method requires optimization from established proteomic-based methods.

Despite the theoretical benefits of increased peak capacity, the system tested requires a number of settings to be optimized before MS comparable peptide identification rates can be achieved. We have found the use of drift time specific collision energies and dynamic range extension settings are crucial to producing optimal HDX-IM-MS results. Importantly, at the sample complexities included in this study, we observe that the complete benefits of IMS are not evident until after manual curation. This combined with a lack of comprehensive information on HDX-IM-MS optimization has likely contributed to the seemingly slow pace at which the methodology has been adopted, despite the potential benefits. Such benefits are almost certain to be amplified for samples of higher complexity than those included in this study, although, this is beyond the scope of this manuscript. Our findings represent favorable levels of improvement in spatial resolution compared with previous HDX-MS analytical developments, with shorter analysis times and no requirement for changes in experimental setup required [14].

As developments in ion mobility and its integration within DIA acquisition workflows continue, the benefits of IMS can only increase. Indeed, the publication of a recent novel multi-mode acquisition (MMA) method [44], which relies upon ion mobility for improved spectral alignment, promises to dramatically improve peptide identification rates for complex samples. The application of MMA to HDX-MS analyses is yet to be realized but it would seem the future of HDX-IM-MS analyses is promising.

## Electronic supplementary material

Below is the link to the electronic supplementary material.

Supplementary Figure 1 (PDF 185 kb)

Supplementary Figure 2 (PDF 371 kb)

Supplementary Figure 3 (PDF 63 kb)

Supplementary Figure 4 (PDF 167 kb)

Supplementary Figure 5 (PDF 198 kb)

Supplementary Data (DOCX 18 kb)
