# Supplementary material for: Online Hydrogen-Deuterium Exchange Traveling Wave Ion Mobility Mass Spectrometry (HDX-IM-MS): a Systematic Evaluation
Source: J Am Soc Mass Spectrom. 2017 Apr 3;28(6):1192–202. doi: 10.1007/s13361-017-1633-z (PMC5438439; doi:10.1007/s13361-017-1633-z)
Supplement: Supplementary file 1 — (PDF 185 kb) [file 13361_2017_1633_MOESM1_ESM.pdf]

a)

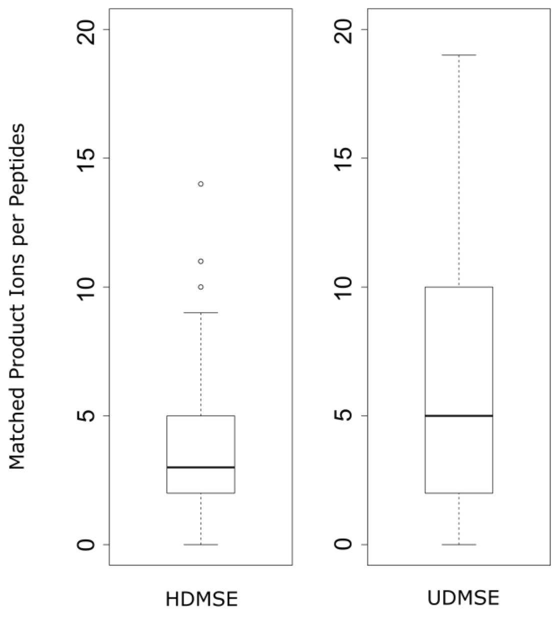

b)

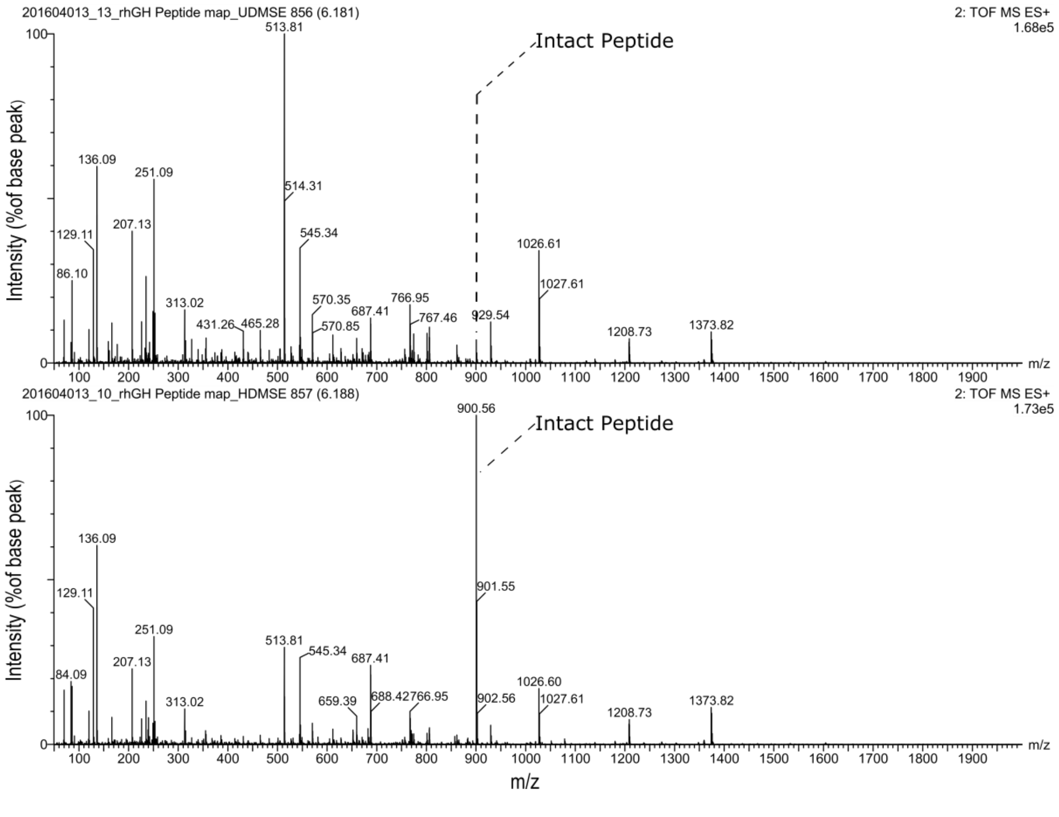

Supplementary Figure 1. UDMS^E peptide fragmentation efficiency is higher than HDMS^E. a) Boxplots of the number of matched product ions for rhGH proteolytic peptides, as identified by HDMS^E or UDMS^E analysis. b) Comparison of peptide high energy spectra acquired by HDMS^E and UDMS^E modes. Data has been combined over the same retention time window.
