# Supplementary material for: Online Hydrogen-Deuterium Exchange Traveling Wave Ion Mobility Mass Spectrometry (HDX-IM-MS): a Systematic Evaluation
Source: J Am Soc Mass Spectrom. 2017 Apr 3;28(6):1192–202. doi: 10.1007/s13361-017-1633-z (PMC5438439; doi:10.1007/s13361-017-1633-z)
Supplement: Supplementary file 2 — (PDF 371 kb) [file 13361_2017_1633_MOESM2_ESM.pdf]

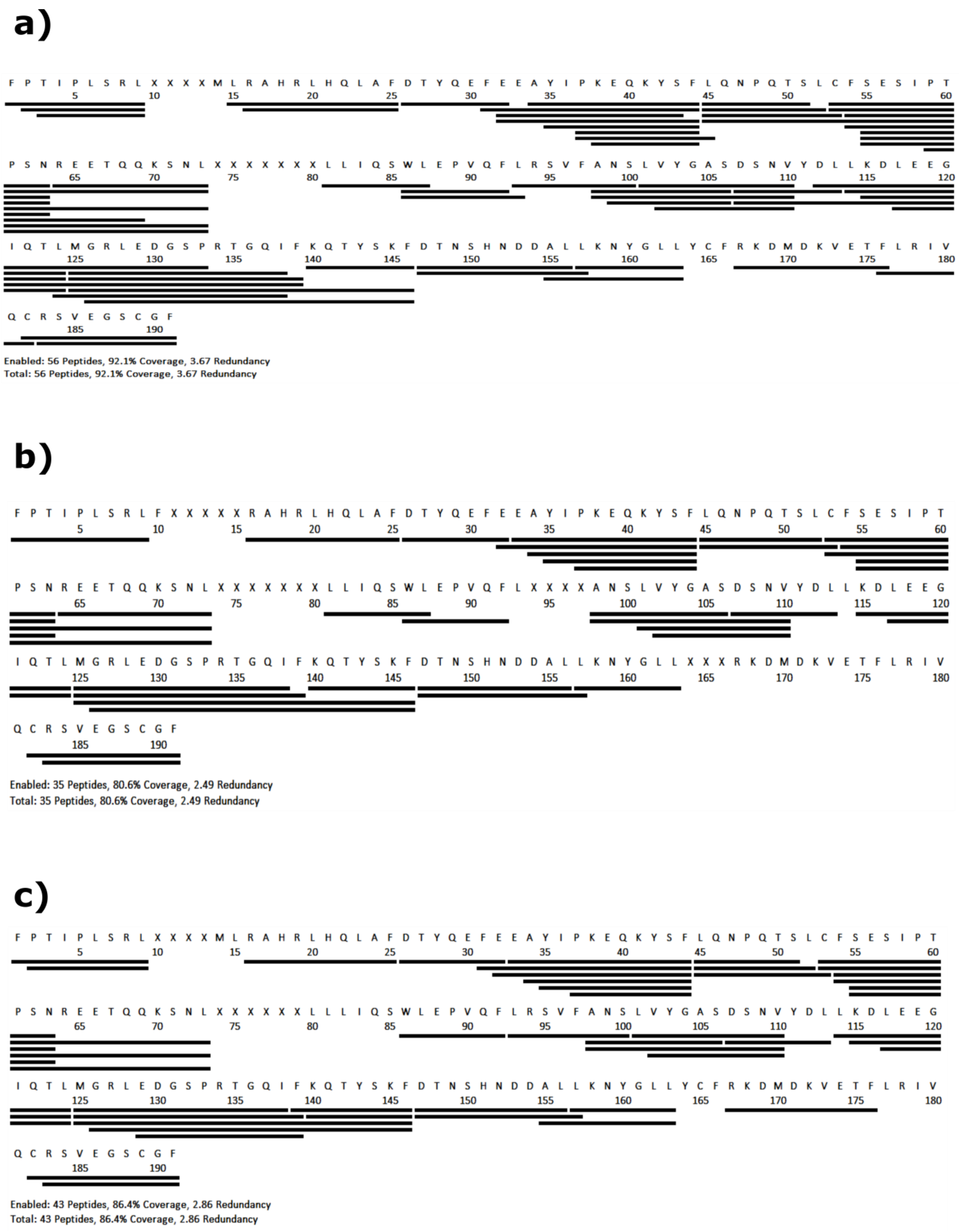

Supplementary Figure 2. rhGH peptide maps generated by MS^E (a) $HDMS^E$ (b) and $UDMS^E$ (c) modes of acquisition.
