# Supplementary material for: Online Hydrogen-Deuterium Exchange Traveling Wave Ion Mobility Mass Spectrometry (HDX-IM-MS): a Systematic Evaluation
Source: J Am Soc Mass Spectrom. 2017 Apr 3;28(6):1192–202. doi: 10.1007/s13361-017-1633-z (PMC5438439; doi:10.1007/s13361-017-1633-z)
Supplement: Supplementary file 3 — (PDF 63 kb) [file 13361_2017_1633_MOESM3_ESM.pdf]

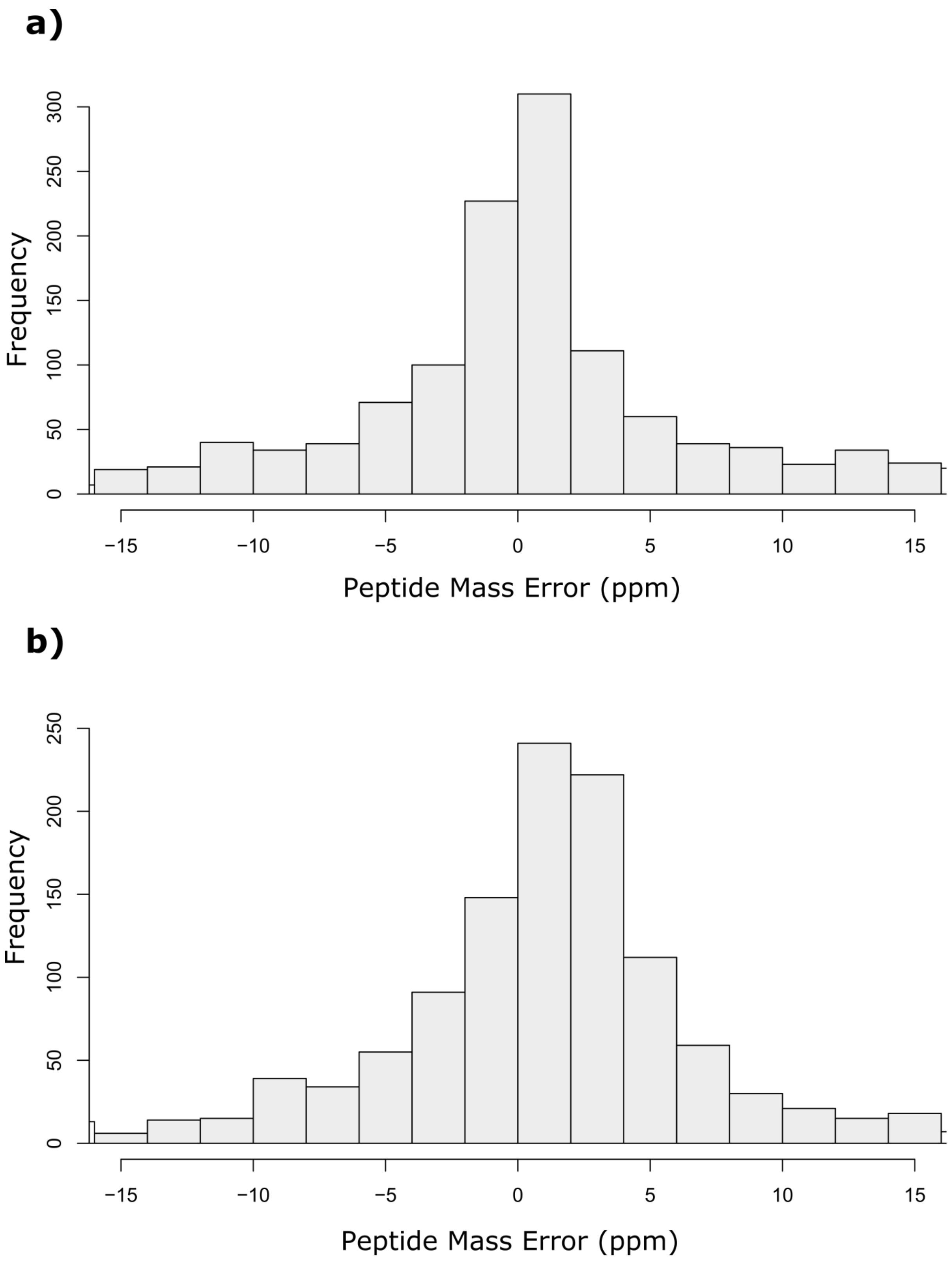

Supplementary Figure 3. Detector saturation when using UDMS^E analysis for peptide map generation causes a small positive mass bias. Shown are histograms of mass errors exported from PLGS derived rhGH peptide identifications. Shown are MS^E (a) and UDMS^E (b) acquisition results.
