# Supplementary material for: Online Hydrogen-Deuterium Exchange Traveling Wave Ion Mobility Mass Spectrometry (HDX-IM-MS): a Systematic Evaluation
Source: J Am Soc Mass Spectrom. 2017 Apr 3;28(6):1192–202. doi: 10.1007/s13361-017-1633-z (PMC5438439; doi:10.1007/s13361-017-1633-z)
Supplement: Supplementary file 4 — (PDF 167 kb) [file 13361_2017_1633_MOESM4_ESM.pdf]

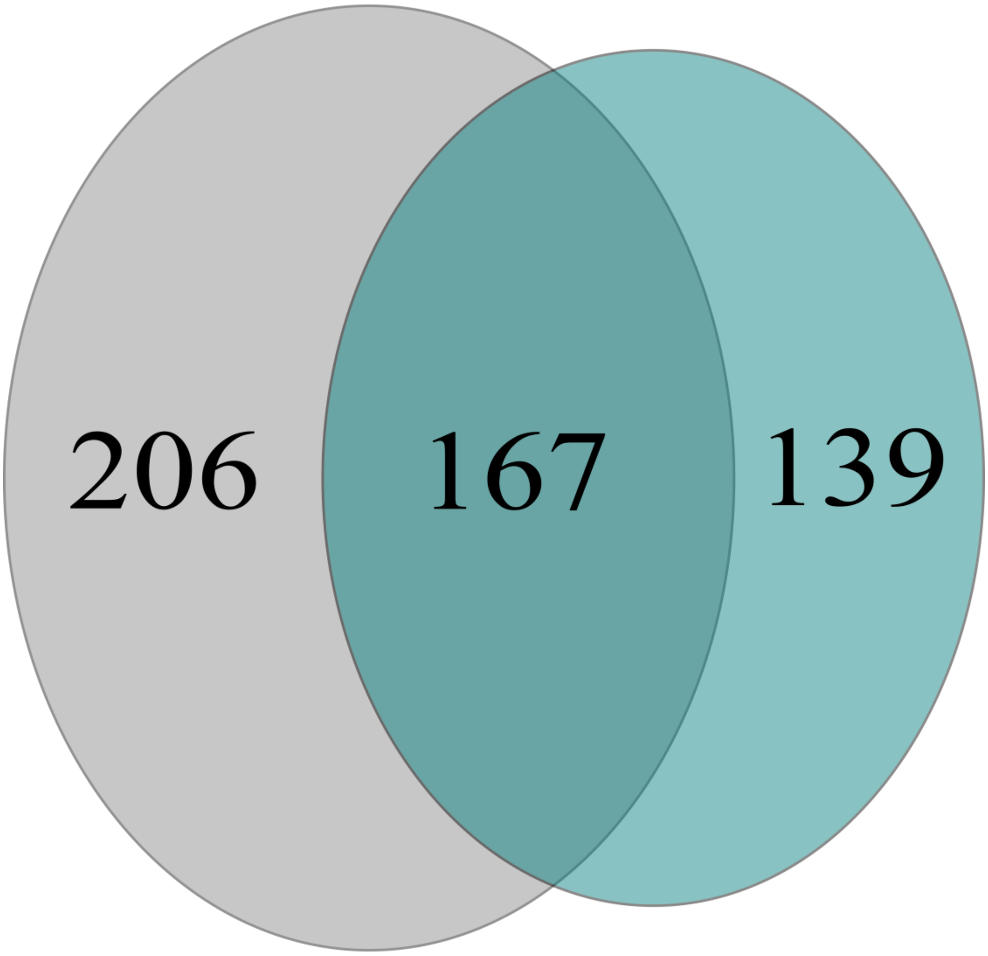

Supplementary Figure 4. Venn diagram of all peptides identified from the analysis of Transferrin + BSA samples by MS^E and $UDMS^E$ modes of acquisition. Peptides included were retained after filtering for replication, the presence of fragment ions and mass accuracy. MS^E and $UDMS^E$ are denoted by teal and grey respectively.
