# Supplementary material for: Online Hydrogen-Deuterium Exchange Traveling Wave Ion Mobility Mass Spectrometry (HDX-IM-MS): a Systematic Evaluation
Source: J Am Soc Mass Spectrom. 2017 Apr 3;28(6):1192–202. doi: 10.1007/s13361-017-1633-z (PMC5438439; doi:10.1007/s13361-017-1633-z)
Supplement: Supplementary file 5 — (PDF 198 kb) [file 13361_2017_1633_MOESM5_ESM.pdf]

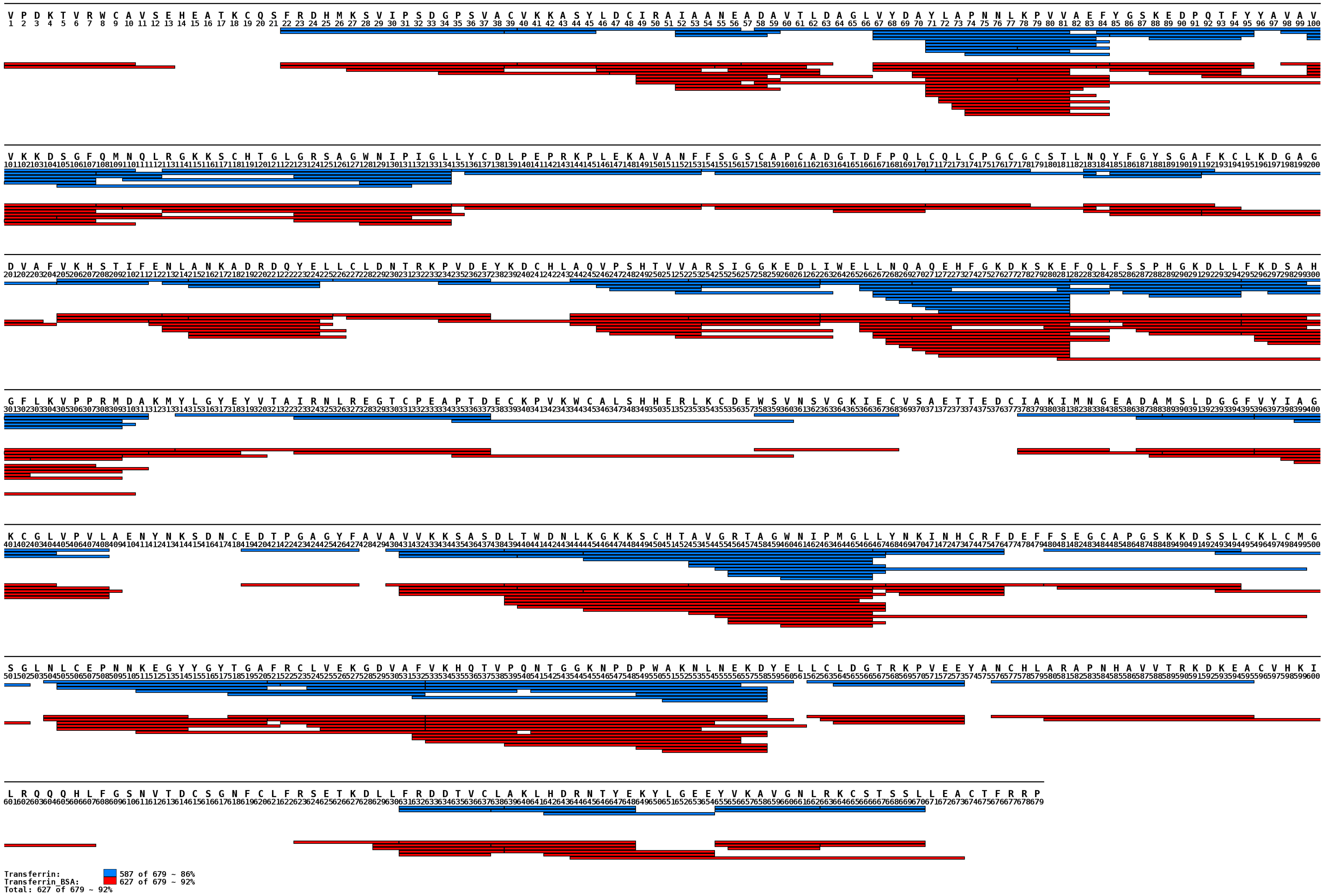
V P D K T V R W C A V S E H E A T K C Q S F R D H M K S V I P S D G P S V A C V K K A S Y L D C I R A I A A N E A D A V T L D A G L V Y D A Y L A P N N L K P V V A E F Y G S K E D P Q T F Y Y A V A V
V K K D S G F Q M N Q L R G K K S C H T G L G R S A G W N I P I G L L Y C D L P E P R K P L E K A V A N F F S G S C A P C A D G T D F P Q L C Q L C P G C G C S T L N Q Y F G Y S G A F K C L K D G A G
D V A F V K H S T I F E N L A N K A D R D Q Y E L L C L D N T R K P V D E Y K D C H L A Q V P S H T V V A R S I G G K E D L I W E L L N Q A Q E H F G K D K S K E F Q L F S S P H G K D L L F K D S A H
G F L K V P P R M D A K M Y L G Y E Y V T A I R N L R E G T C P E A P T D E C K P V K W C A L S H H E R L K C D E W S V N S V G K I E C V S A E T T E D C I A K I M N G E A D A M S L D G G F V Y I A G
K C G L V P V L A E N Y N K S D N C E D T P G A G Y F A V A V V K K S A S D L T W D N L K G K K S C H T A V G R T A G W N I P M G L L Y N K I N H C R F D E F F S E G C A P G S K K D S S L C K L C M G
S G L N L C E P N N K E G Y Y G Y T G A F R C L V E K G D V A F V K H Q T V P Q N T G G K N P D P W A K N L N E K D Y E L L C L D G T R K P V E E Y A N C H L A R A P N H A V V T R K D K E A C V H K I
L R Q Q Q H L F G S N V T D C S G N F C L F R S E T K D L L F R D D T V C L A K L H D R N T Y E K Y L G E E Y V K A V G N L R K C S T S S L L E A C T F R R P
Transferrin: 587 of 679 ~ 86%
Transferrin_BSA: 627 of 679 ~ 92%
Total: 627 of 679 ~ 92%

Supplementary Figure 5. Overlaid Transferrin sequence coverage maps (after manual validation of time-course data) when acquiring data in MS^E mode (blue) or using the combined approach (red). Data is from the analysis of transferrin + BSA samples.
