# Supplementary material for: Online Hydrogen-Deuterium Exchange Traveling Wave Ion Mobility Mass Spectrometry (HDX-IM-MS): a Systematic Evaluation
Source: J Am Soc Mass Spectrom. 2017 Apr 3;28(6):1192–202. doi: 10.1007/s13361-017-1633-z (PMC5438439; doi:10.1007/s13361-017-1633-z)
Supplement: Supplementary file 6 — (DOCX 18 kb) [file 13361_2017_1633_MOESM6_ESM.docx]

# This script accepts the state data .csv output from DynamX. Includes all main data processing steps required for data analysis

# Outputs an excel spreadsheet containing all data and calculations

# Load in StateData.csv file from DynamX as "df"

library(xlsx)

# define the two condition you are comparing and the deuteration time points you used

Condition1 <- "ProteinA"

Condition2 <- "ProteinA_B"

Time1 <- 0.00

Time2 <-2.50

Time3 <- 60

Time4 <- 240

ExcelFileName <- "Name.xlsx"

TimeA <- toString(Time1)

TimeB <- toString(Time2)

TimeC <- toString(Time3)

TimeD <- toString(Time4)

# initial calculations of exchange, standard deviation etc

df$PercentageExchange <- df$Uptake/df$MaxUptake

df$SDExchange <- (df$Uptake.SD/df$Uptake)*df$PercentageExchange

df$rsd <- df$SDExchange/df$PercentageExchange

df[is.na(df)] <- 0

df$Exposure <- round(df$Exposure, digits=2)

State1 <- subset(df, State == Condition1) # Split the two conditions into seperate data frames

State2 <- subset(df, State == Condition2) # STATE NAMES WILL NEED TO BE CHANGED TO MATCH THE DATA THAT HAS BEEN EXPORTED

# Subset data for the inital excel spreadsheet

# WILL NEED TO BE ALTERED IS TIMINGS ARE DIFFERENT

State1_T1 <- subset(State1, Exposure== Time1)

State1_T2 <- subset(State1, Exposure== Time2)

State1_T3 <- subset(State1, Exposure== Time3)

State1_T4 <- subset(State1, Exposure== Time4)

State2_T1 <- subset(State2, Exposure== Time1)

State2_T2 <- subset(State2, Exposure== Time2)

State2_T3 <- subset(State2, Exposure== Time3)

State2_T4 <- subset(State2, Exposure== Time4)

# Function to combine the data frames into an initial excel spreadsheet

# NAME OF SPREADSHEET AND TIMINGS CAN BE CHANGED TO SUIT THE DATA

write.xlsx(State1_T1, file=ExcelFileName, sheetName=paste(Condition1,"T",TimeA, sep=""))

write.xlsx(State1_T2, file=ExcelFileName, sheetName=paste(Condition1,"T",TimeB, sep=""), append=TRUE)

write.xlsx(State1_T3, file=ExcelFileName, sheetName=paste(Condition1,"T",TimeC, sep=""), append=TRUE)

write.xlsx(State1_T4, file=ExcelFileName, sheetName=paste(Condition1,"T",TimeD, sep=""), append=TRUE)

write.xlsx(State2_T1, file=ExcelFileName, sheetName=paste(Condition2,"T",TimeA, sep=""), append=TRUE)

write.xlsx(State2_T2, file=ExcelFileName, sheetName=paste(Condition2,"T",TimeB, sep=""), append=TRUE)

write.xlsx(State2_T3, file=ExcelFileName, sheetName=paste(Condition2,"T",TimeC, sep=""), append=TRUE)

write.xlsx(State2_T4, file=ExcelFileName, sheetName=paste(Condition2,"T",TimeD, sep=""), append=TRUE)

State1_T1 <- subset(State1, Exposure== Time1, select = c(Protein,Start,End,Sequence,MaxUptake, Uptake, Uptake.SD, rsd)) # Subset data for summary peptide data frame only keeping select columns.

State1_T2 <- subset(State1, Exposure== Time2, select = c(Protein,Start,End,Sequence,MaxUptake, Uptake, Uptake.SD, rsd))

State1_T3 <- subset(State1, Exposure== Time3, select = c(Protein,Start,End,Sequence,MaxUptake, Uptake, Uptake.SD, rsd))

State1_T4 <- subset(State1, Exposure== Time4, select = c(Protein,Start,End,Sequence,MaxUptake, Uptake, Uptake.SD, rsd))

State2_T1 <- subset(State2, Exposure== Time1, select = c(Protein,Start,End,Sequence,MaxUptake, Uptake, Uptake.SD, rsd)) # Subset data for summary peptide data frame only keeping select columns.

State2_T2 <- subset(State2, Exposure== Time2, select = c(Protein,Start,End,Sequence,MaxUptake, Uptake, Uptake.SD, rsd))

State2_T3 <- subset(State2, Exposure== Time3, select = c(Protein,Start,End,Sequence,MaxUptake, Uptake, Uptake.SD, rsd))

State2_T4 <- subset(State2, Exposure== Time4, select = c(Protein,Start,End,Sequence,MaxUptake, Uptake, Uptake.SD, rsd))

# merge data ready for calculations

# Will throw an error up but this is only due to the program naming some of the columns the same thing due to a lack of options

l <- list(State1_T1,State1_T2,State1_T3,State1_T4)

summary_control_peptide <- l[[1]]

for (i in 2:length(l)) {

summary_control_peptide <- merge(summary_control_peptide,l[[i]], by=c("Protein","Start","End","Sequence","MaxUptake"))

}

l <- list(State2_T1,State2_T2,State2_T3,State2_T4)

summary_condition_peptide <- l[[1]]

for (i in 2:length(l)) {

summary_condition_peptide <- merge(summary_condition_peptide,l[[i]], by=c("Protein","Start","End","Sequence","MaxUptake"))

}

#NEED TO RENAME COLUMNS SO THAT THEY CAN BE UNDERSTOOD

colnames(summary_control_peptide)[6:17] <- c(paste("T",TimeA, " ", "Uptake", sep=""), paste("T",TimeA, " ", "SDxchange", sep=""), paste("T",TimeA, " ", "rsd", sep="" ), paste("T",TimeB, " ", "Uptake", sep=""), paste("T",TimeB, " ", "SDxchange", sep=""), paste("T",TimeB, " ", "rsd", sep=""), paste("T",TimeC, " ", "Uptake", sep=""), paste("T",TimeC, " ", "SDxchange", sep=""), paste("T",TimeC, " ", "rsd", sep="" ), paste("T",TimeD, " ", "Uptake", sep=""), paste("T",TimeD, " ", "SDxchange", sep=""), paste("T",TimeD, " ", "rsd", sep="" ))

colnames(summary_condition_peptide)[6:17] <- c(paste("T",TimeA, " ", "Uptake", sep=""), paste("T",TimeA, " ", "SDxchange", sep=""), paste("T",TimeA, " ", "rsd", sep="" ), paste("T",TimeB, " ", "Uptake", sep=""), paste("T",TimeB, " ", "SDxchange", sep=""), paste("T",TimeB, " ", "rsd", sep=""), paste("T",TimeC, " ", "Uptake", sep=""), paste("T",TimeC, " ", "SDxchange", sep=""), paste("T",TimeC, " ", "rsd", sep="" ), paste("T",TimeD, " ", "Uptake", sep=""), paste("T",TimeD, " ", "SDxchange", sep=""), paste("T",TimeD, " ", "rsd", sep="" ))

# Need to reorder the merge data which has been jumbled up

summary_control_peptide <- summary_control_peptide[order(summary_control_peptide[,2],summary_control_peptide[,3]),]

summary_condition_peptide <- summary_condition_peptide[order(summary_condition_peptide[,2],summary_condition_peptide[,3]),]

write.xlsx(summary_control_peptide, file=ExcelFileName, sheetName="Summary_control_peptide", append=TRUE)

write.xlsx(summary_condition_peptide, file=ExcelFileName, sheetName="Summary_condition_peptide", append=TRUE)

# calculate degree of differential exchange between control and condition

DifferentialExchange <- summary_control_peptide[,1:4]

DifferentialExchange$Time1 <- summary_control_peptide[,6]-summary_condition_peptide[,6]

DifferentialExchange$Time2 <- summary_control_peptide[,9]-summary_condition_peptide[,9]

DifferentialExchange$Time3 <- summary_control_peptide[,12]-summary_condition_peptide[,12]

DifferentialExchange$Time4 <- summary_control_peptide[,15]-summary_condition_peptide[,15]

DifferentialExchange$Abs_Time1 <- abs(DifferentialExchange$Time1)

DifferentialExchange$Abs_Time2 <- abs(DifferentialExchange$Time2)

DifferentialExchange$Abs_Time3 <- abs(DifferentialExchange$Time3)

DifferentialExchange$Abs_Time4 <- abs(DifferentialExchange$Time4)

DifferentialExchange$Time1_Uncertainty <- ((summary_control_peptide[,7]^2)+(summary_condition_peptide[,7]^2))^0.5

DifferentialExchange$Time2_Uncertainty <- ((summary_control_peptide[,10]^2)+(summary_condition_peptide[,10]^2))^0.5

DifferentialExchange$Time3_Uncertainty <- ((summary_control_peptide[,13]^2)+(summary_condition_peptide[,13]^2))^0.5

DifferentialExchange$Time4_Uncertainty <- ((summary_control_peptide[,16]^2)+(summary_condition_peptide[,16]^2))^0.5

# renaming of differential columns to match the input of timings

colnames(DifferentialExchange) <- c("protein", "Start", "End", "Sequence",paste("Time",TimeA, " ", "Diff", sep=""), paste("Time",TimeB, " ", "Diff", sep=""),paste("Time",TimeC, " ", "Diff", sep=""),paste("Time",TimeD, " ", "Diff", sep=""), paste("Time",TimeA, " ", "Abs Diff", sep=""), paste("Time",TimeB, " ", "Abs Diff", sep=""),paste("Time",TimeC, " ", "Abs Diff", sep=""),paste("Time",TimeD, " ", "Abs Diff", sep=""),paste("Time",TimeA, " ", "Uncertainty", sep=""), paste("Time",TimeB, " ", "Uncertainty", sep=""),paste("Time",TimeC, " ", "Uncertainty", sep=""),paste("Time",TimeD, " ", "Uncertainty", sep=""))

DifferentialExchange$SumofUncertainties <- (DifferentialExchange[,14]^2 + DifferentialExchange[,15]^2 + DifferentialExchange[,16]^2)^0.5

DifferentialExchange$SumofDifferential <- rowSums(DifferentialExchange[,10:12])

DifferentialExchange$Average_of_Uncertainties <- rowMeans(DifferentialExchange[,14:16])

DifferentialExchange$IRank <- 1:nrow(DifferentialExchange)

write.xlsx(DifferentialExchange, file=ExcelFileName, sheetName="differential", append=TRUE)

#This script takes an input of two different differential files (which is created using the previous script) and combines the data. Where the duplicate peptide HDX data is present the data with the lowest measurement uncertainty is retained. Data combined is usually from a MSE and a UDMSe HDX run but can be any combination of HDX data.

MSE$Method <- replicate(nrow(MSE),"MSE")

UDMSE$Method <- replicate(nrow(UDMSE),"UDMSE") #creates a column with reference to the method of acquistion

df <- rbind(MSE,UDMSE) # combines the rows from the two differential file to create a master data frame

df.agg <- aggregate(Average_of_Uncertainties~Sequence,df,min) # for each peptide sequence identifies the lowest "Average_of_Uncertainties" value

df.merged <- merge(df.agg,df, by=c("Sequence","Average_of_Uncertainties")) # Creates final merged data frame retaining the data with the lowest measurment uncertainty

df.merged <- df.merged[order(df.merged[,5],df.merged[,6]),] #reorders final data set based on sequence position of peptides

write.csv(df.merged, file=CSVFileName)
